# Does displacement of lower pole stones during retrograde intrarenal surgery improves stone-free status? A systematic review and meta-analysis

**DOI:** 10.1590/acb386623

**Published:** 2023-12-01

**Authors:** Roberto Nogueira Santana, Breno Cordeiro Porto, Carlo Camargo Passerotti, Everson Luiz de Almeida Artifon, José Pinhata Otoch, José Arnaldo Shiomi da Cruz

**Affiliations:** 1 Universidade Nove de Julho – Surgery Department – São Bernardo do Campo (SP) – Brazil.; 2 Universidade de São Paulo – School of Medicine – Surgical Technique and Experimental Surgery – São Paulo (SP) – Brazil.; 3 Hospital Alemão Oswaldo Cruz – Urology Department – São Paulo (SP) – Brazil.

**Keywords:** Lithotripsy, Kidney Calculi, Ureteroscopy

## Abstract

**Purpose:**

Kidney stones are one of the most common urological diseases worldwide. The size and location of the stone are the most important factors in determining the most suitable treatment options. The aim of this review was to evaluate the displacement of lower pole stones.

**Methods:**

Three studies assessing the efficacy of translocating kidney stones from the lower pole of the kidney to other locations during retrograde intrarenal surgery published in the last 20 years were included. A systematic search was conducted in the PubMed, Embase, Latin American and Caribbean Health Sciences Literature (LILACS), and Web of Science databases using the following search terms: “Lower pole,” “Lithotripsy.” Meta-analysis was performed using Review Manager version 5.4.

**Results:**

Stone-free rates were improved through displacement (odds ratio – OR = -0.15; 95% confidence interval–95%CI -0.24–-0.05; p = 0.002; I2 = 21%), but at the cost of increased surgical duration (mean difference = -12.50; 95%CI -24.06–-0.95; p = 0.03; I2 = 94%). Although this represents a potentially negative outcome, the improvement in clearance rates justifies the additional investment of time and effort.

**Conclusions:**

Displacement of lower pole kidney stones for subsequent lithotripsy brings significant benefits in terms of stone-free rate, with no difference in laser energy usage. However, it results in increased surgical time. Despite these factors, the benefits to patients undergoing the procedure are substantial.

## Introduction

Kidney stones are one of the most common urological diseases worldwide, with an estimated prevalence ranging from 1 to 13% in different regions of the globe^1^^,^ 2. The number of people affected by the disease continues to grow every year^3^. Complications include acute renal failure secondary to obstruction, anuria, urinary tract infection with renal obstruction, and sepsis^4^.

The size and location of the stone are the most important factors in determining which treatment options are the most appropriate, but the surgeon’s treatment preference is also important in making treatment decisions for each case^5^. In patients who do not require urgent surgery and have an indication for planned stone removal, the choice of surgical procedure depends primarily on the size and location of the stones, but it can also be influenced by other patient’s characteristics, such as the anatomy of the urinary tract or stone composition, as well as associated conditions like obesity and bleeding diathesis^3^^,^ 6. Regarding that, the lower pole has more challenging access due to the inherent anatomy of the kidneys and upper urinary tract. Therefore, choosing to move the renal calculus from the lower pole to another area provides the surgeon with improved visualization and easy access during the procedure, thus enabling a more effective surgery and reducing additional damage.

The recommended size of stones treated by ureteroscopy for retrograde intrarenal surgery (RIRS) increases with each new guideline update^7^. The current cutoff is 20 mm or larger, favoring a percutaneous approach in those cases. Therefore, there is a need for a well-described study that comprehensively evaluates how this translocation can help increase the stone-free rate (SFR) and diminish complications in patients undergoing RIRS.

Thus, the purpose of this study was to conduct a meta-analysis of studies that assessed the improvement of SFR in displacement of lower pole stones during retrograde intrarenal surgery.

## Methods

### Eligibility

A search was conducted at PubMed, Embase, Latin American and Caribbean Health Sciences Literature (LILACS), and Web of Science databases from its inception to July 2023 to identify trials reporting possible improvement in displacement of lower pole stones during RIRS. We included: adults (>= 18 years old) submitted to RIRS for calculi in the lower pole of the kidney. We excluded:

- Patients with less than 18 years old;
- Patients undergoing a different approach than RIRS;
- Patients submitted to RIRS for other stones in other poles of the kidney or in the renal pelvis.

### Search strategy

The search strategy included terms related to the intervention “Lithotripsy” and terms related to “Lower pole”. This study was registered at PROSPERO (CRD42023422564).

### Endpoints

Our primary outcome of interest is the effectiveness of translocating lower pole renal stones to other locations, such as the upper pole or interpolar region during RIRS. As second outcomes of interest, we analyzed the operative time, energy laser use and complications^8^.

### Screening

The duplicates (n = 611) were removed using Endnote online 20. Potentially relevant studies were selected for full-text assessment after two independent researchers (RS and BP) screened the studies by title and abstract, and disagreements were solved by a third one (JA).

### Data extraction and risk of bias

Two independent researchers (RS and BP) extracted the data based on a predefined protocol and disagreements were solved by a third one (JA). Two authors independently extracted the data following predefined search criteria and quality assessment. The Review Manager 5.4 (Cochrane Center, The Cochrane Collaboration, Denmark) was used to assess the quality of the studies.

A standardized data extraction form was used to capture demographic data, such as gender, age, and body mass index (BMI). Data regarding the stones themselves, including size in millimeters, stone laterality, and SFR were also recorded. Information on surgical equipment, including the ureteroscopes used and their type, diameter, and brand, as well as details about lithotripsy, laser type, laser brand, fiber size, duration, and fragmentation configuration were captured. Additionally, data on author(s), publication year, study design, sample size, outcome measures, main results, and reported effect measures were collected.

### Statistical analysis

This systematic review and meta-analysis were performed and reported in accordance with the Cochrane Collaboration Handbook for Systematic Review of interventions and the Preferred Reporting Items for Systematic Reviews and Meta-Analysis (PRISMA) Statement guidelines.

Continuous outcomes are presented as a mean difference (MD) with 95% confidence interval (95%CI). Dichotomous data are presented as relative risk (RR), standardized mean difference (SMD), and their 95%CI were calculated as effect sizes.

Subgroup analysis was conducted to explore the source of heterogeneity observed among the studies. Subgroups were defined based on participants’ BMI, age, and stone size.

Pooled estimates were calculated with the random-effects model, considering that the patients came from different populations.

## Results

**Figure 1:**
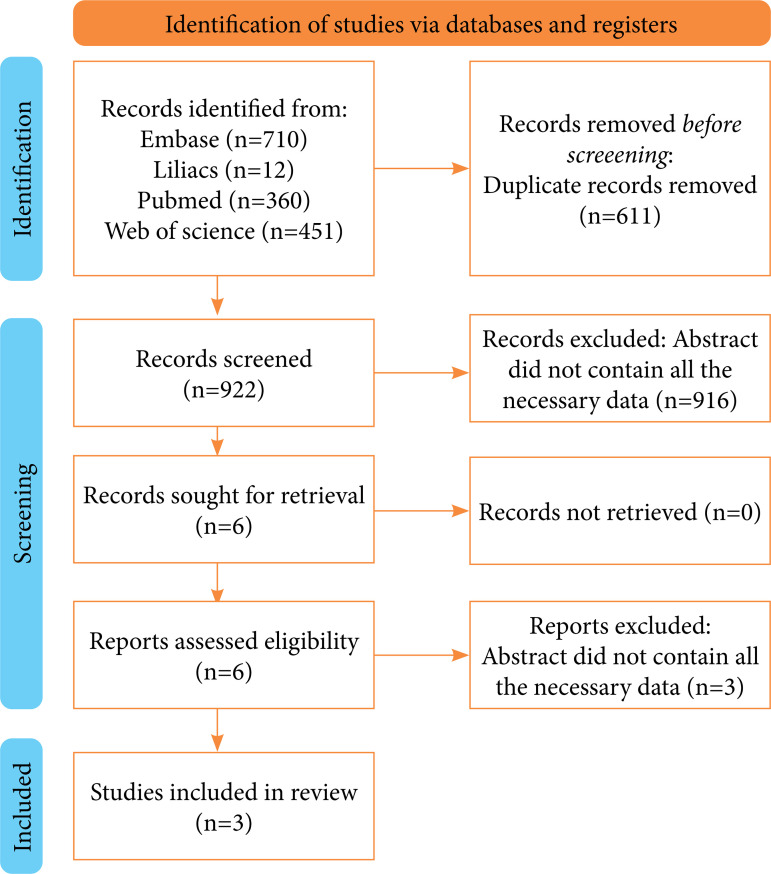
PRISMA flowchart.

Source: Elaborated by the authors.

Our search retrieved 1,533 studies, of which three randomized controlled trials were included (Fig. 1). Table 1 describes the baseline characteristics of included studies, which were Yaghoubian et al.^9^, Shrestha et al.^10^, and Gallante et al.^11^.

**Table 1:** Baseline characteristics of patients of included studies*.

Study	Type of study	Language	Methods	No. of patients	Sex		Age (median)	BMI (median)	ASA (median)
					Male n (%)	Female n (%)			
Yaghoubian et al.^9^	Prospective randomized trial	English	Intervention	62	39 (57)	23 (43)	57 (51;64)	27.5 (24.1;31.7)	2
			Control	62	30 (44)	32 (56)	58 (47;68)	28.7 (25.2;33.1)	2
Shrestha et al.^10^	Prospective randomized trial	English	Intervention	33	26 (78.8)	7 (21.2)	42.0 ± 13.3	24.08+–3.89	NA
			Control	35	22 (62.9)	13 (37.1)	32.88 ± 12.03	23.82+ –3.43	NA
Gallante et al.^11^	Prospective randomized trial	English	Intervention	39	17 (43.6)	22 (56.4)	62 (56;70)	28.7 (24.0;34.2)	2
			Control	29	8 (27.6)	2172.4)	57 (45;69)	28.5 (24.4;33.3)	2

* Continuous data are presented as median ± standard deviation and n (%); BMI: body mass index; ASA: American Society of Anesthesiologists; NA: not available. Source: Elaborated by the authors.

The total sample size of the included studies was 260 patients, 134 from the intervention group, and 126 from the control group. These patients were reassessed a few weeks after surgery to analyze the SFR following RIRS with the stone being moved from the lower pole in the intervention group. The SFR was evaluated after four weeks using kidney, ureter, and bladder radiography or ultrasonography.

The mean age of all patients included were 51.5 years old, all previously diagnosed with renal calculi smaller than 2 cm in the lower pole. The mean BMI was 26.9 kg/m^2^, with average male participants in the intervention group (82; 61.19%) and female participants in the control group (60; 47.61%). The focus was on the displacement of renal calculi from the lower pole to other locations, either in the upper pole or interpolar region. Both the intervention and control groups underwent RIRS.

In terms of complications, which were measured based on Clavien-Dindo grades, it was seen 11.3% complications grade 1/2 in intervention group and 4.8% in control group in Yaghoubian et al.’s study^9^. On the other hand, the Shrestha et al.’s^10^ trial presented 6% of grade 1/2 complications in the intervention branch and 2.8% in the control branch. In Gallante et al.’s study^11^, the complication rate was not available. Also, it was not seen Clavien-Dindo grades 3/4 in any study here included (Table 2).

**Table 2:** Stone-free rate and complications rate.

Study	Methods	Patients with stone-free (No.)	Stone-free rate (%)	Operative time (min)	Ureteral access sheath used–Nº (%)	Stone hounsfield units (median)	Complications (No.)	
							Clavien I-II	Clavien III-IV
Yaghoubian et al.^9^	Intervention	59	95	65	24 (35)	924	7	0
	Control	46	74	55	13(19)	868	3	0
Shrestha et al.^10^	Intervention	30	91	48.3*	27* (81.8)	1,102.97*	2	0
	Control	30	85.7	42.6*	26* (74.3)	966.42*	1	0
Gallante et al.^11^	Intervention	38	97.4	77.5	17 (43.6)	NA	NA	NA
	Control	24	82.8	53	6 (21.4)	NA	NA	NA

* Mean;

NA: not available. Source: Elaborated by the authors.

**Figure 2:**
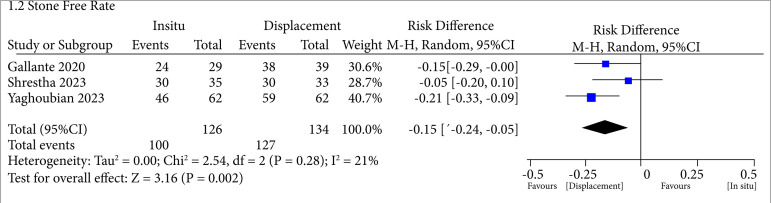
Increased stone-free rate when displacing the stone from lower pole to another location.

**Figure 3:**
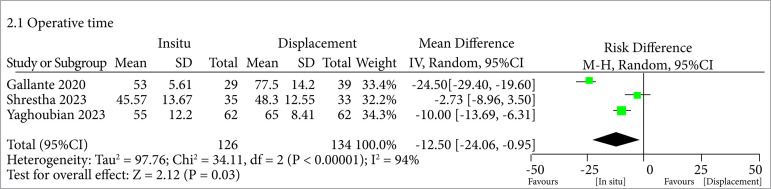
Higher operative time when displacing the stone from lower pole to another location

In terms of SFR, it was higher when the displacement was done for lower pole stones (odds ratio – OR = -0.15; CI95 -0.24–-0.05; p = 0.002; I2 = 21%) (Fig. 2). Although, regarding the operative time, we could see that it was also higher in the displacement group (MD = 12.50; 95%CI95 -24.06–-0.95; p = 0.03; I2 = 94%) (Fig. 3). So, in general, we could see an increased SFR among patients who underwent stone displacement compared to the control group, with an average SFR of 97.47 and 80.83%, respectively.

95%CI: 95% confidence interval; df: degrees of freedom. Source: Elaborated by the authors.

95%CI: 95% confidence interval; df: degrees of freedom. Source: Elaborated by the authors.

Also, the SFR analysis yielded a Tau^2^ value of 0, indicating minimal variability between the studies. The heterogeneity among the studies resulted in a χ^2^ value of 2.54 with 2 degrees of freedom (df) and p = 0.28, suggesting no statistically significant evidence of heterogeneity. The proportion of total variability, as indicated by an I^2^ value of 21%, suggested low heterogeneity among the included studies. In the overall effect test, this meta-analysis showed a Z value of 3.16 with a corresponding p = 0.002, indicating statistically significant evidence of an overall effect.

**Figure 4:**
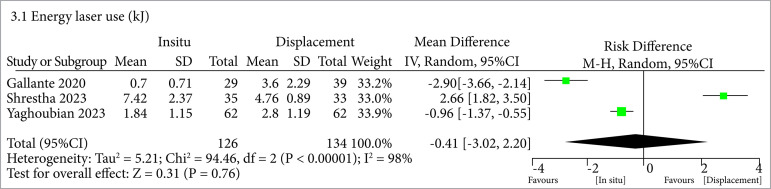
No difference regarding the energy laser use between the two approaches.

SD: standard deviation; 95%CI: 95% confidence interval; df: degrees of freedom. Source: Elaborated by the authors.

No difference was found when comparing the energy laser use between the two approaches (MD = -0.41; 95%CI -3.02–2.20; p = 0.76; I2 = 98%) (Fig. 4, Table 3).

**Figure 5:**
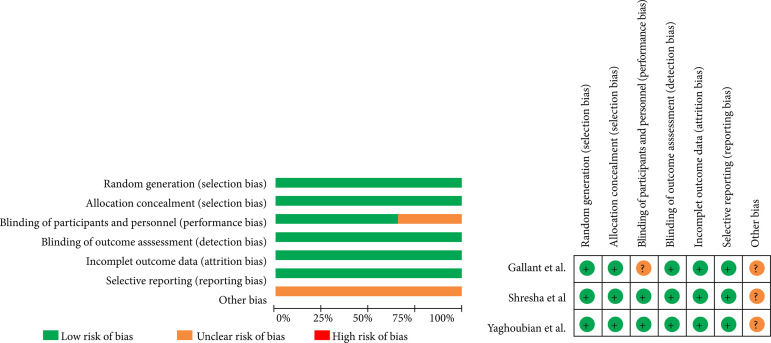
Risk of bias assessment.

Source: Elaborated by the authors.

The articles here included presented an overall moderate bias, as assessed by Review Manager 5.4. Moreover, the study conducted by Gallante et al.^11^ demonstrated a higher degree of bias when compared to others, due to the presence of a performance bias (Fig. 5).

**Table 3:** Lithotripsy data and equipment used.

Study	Methods	Lithotripsy				
		Laser type	Laser brand	Fiber size	Duration	Fragmentation configuration (energy in Joules and frequency in Hertz)
Yaghoubian et al.^10^	Intervention	Holmium of 120 W with Moses technology	Lumenis Pulse, Boston Scientific	200 micrometers	Until all stones were fragmented into small particles	0.5 J and 5 Hz
	Control					
Shrestha et al.^11^	Intervention	Holmium:YAG	Lumenis Inc	200 micrometers	Until the dust floated or the fragments could be easily removed with gentle irrigation pressure	0.8–1 J and 8–10 Hz
	Control					
Gallante et al.^12^	Intervention	NA	NA	NA	NA	NA
	Control	NA	NA	NA	NA	NA

NA: not available. Source: Elaborated by the authors. Source: Elaborated by the authors.

## Discussion

Synthesized from a compilation of diverse studies, this comprehensive review and meta-analysis investigates whether SFR can be enhanced through the displacement of lower pole stones during RIRS^12^^,^ 13. Our exploration yielded invaluable insights regarding the clinical significance and effectiveness of stone displacement during RIRS surgery.

During our analysis, a pivotal discovery emerged: the intervention group exhibited a notably elevated SFR when compared to the control group, even in the presence of observed heterogeneity^14^^,^ 15. This indicates that the intentional displacement of lower pole stones during RIRS plays a significant role in achieving better outcomes in terms of stone fragmentation and removal, emphasizing the critical nature of stone displacement as a successful treatment approach.

While the augmentation of SFR was achieved through displacement, it is essential to acknowledge the trade-off in the form of an extended surgical duration^16^^,^ 17. The consistent pattern of an extended operative time within the intervention group is evident across all studies included in our analysis, as demonstrated by the following findings: Yaghoubian et al.^9^ (65 vs. 55 min); Shrestha et al.^10^ (48.3 vs. 42.57 min); and finally Gallante et al.^11^ (77.5 vs. 53 min). Therefore, relocating a stone from the lower pole to an alternative site inherently requires more time compared to fragmenting it in its original position and subsequently extracting it^18^^,^ 19. This drawback also introduces the potential for increased resource consumption and places an additional economic burden on the surgical procedure.

Concerning the utilization of laser energy during the surgical procedures, no significant disparities were observed among the examined groups. This finding supports the null hypothesis and further reinforces the safety of the technique for displacing lower pole stones, as it does not contribute to heightened energy consumption.

In terms of heterogeneity, the included studies exhibited certain discrepancies when evaluating this parameter. Variability in patients’ characteristics and equipment utilization could have contributed to the identified heterogeneity in operative time (I2 = 94%) and energy laser use (I2 = 98%). However, the SFR demonstrated a relatively low-heterogeneity rate (21%) and yielded a Tau^2^ value of 0, likely indicating a high degree of uniformity in the employed surgical techniques. These differences should be considered when reviewing the results, and future research efforts should strive to address these causes of heterogeneity to present more stable and accurate evidence.

Yaghoubian et al.^9^ conducted a single center randomized controlled trial with one month follow-up, and were able to indicate that SFR was significantly higher in the intervention group, suggesting a strong advantage for these calculi before laser lithotripsy initiation. However, in this study, when smaller and larger stones were analyzed separately, a significant difference in SFR between the groups was found only for smaller stones, and this difference did not reach statistical significance for larger stones. Furthermore, the examination of the results obtained by the two surgeons revealed that both achieved higher SFR when they displaced the stone, but this difference reached statistical significance only for one of the surgeons, probably due to the smaller sample size analyzed by the other surgeon.

Likewise, Shrestha et al.^10^ conducted a single center randomized controlled trial, which followed their patients for three months. While a general trend toward enhanced SFR was observed in patients undergoing stone relocation followed by lithotripsy (92%) in contrast to in-situ lithotripsy (85.7%), this difference did not achieve statistical significance. There were no differences between the groups concerning operation time, total laser energy used, and laser duration. The similarity in surgical duration, despite additional time required for basketing and relocating the fragments to other poles in the group, could be attributed to ergonomic challenges and effective lithotripsy in the in-situ group. The incidence of complications displayed parallel patterns between the groups, predominantly falling under Clavien grade I, with fever emerging as the most frequent complication. Despite the displacement strategy, residual fragments originating from the lower calyx were detected in two patients, underscoring the continued reliance on the lower calyx region.

Lastly, in Gallante et al.’s study^11^, a prospective randomized trial with a follow-up duration of one month, it was demonstrated that patients with displaced stones exhibited a notably higher SFR when compared to the control group. However, as we could expect, the intervention group experienced longer surgical durations and increased laser energy consumption. Consequently, the study concluded that the displacement of lower pole stones necessitates extended operating room time, with significant improvement in stone elimination rate compared to patients treated in situ.

It is imperative to acknowledge that our analysis is subject to certain limitations that warrant careful consideration^20^^,^ 21. Firstly, the studies encompassed in our analysis were constrained by factors such as a restricted sample size. Secondly, the presence of two different surgeons applying distinct techniques in Yaghoubian et al.’s study^9^ could potentially introduce bias due to procedural variability and a lack of standardization in certain surgical aspects^22^. Thirdly, we could not assess properly the differences in terms of lithotripsy data and equipment used, as Gallante et al.’s study^11^ omitted such data. Fourthly, the employment of imaging methods in Yaghoubian et al.’s^9^ and Shrestha et al.’s^10^ trials possessed lower sensitivity in comparison to computed tomography. This reliance on less sensitive imaging could lead to additional costs for patients in the postoperative period^23^^,^ 24.

Our study does leave certain questions unanswered. One issue pertains to the duration of follow-up. Across all studies included here, the follow-up period was relatively brief. Additionally, an aspect that warrants further investigation is the comparison of equipment utilized^25^^,^ 26. Notably, Yaghoubian et al.^9^ and Shrestha et al.^10^ employed the same laser type but with distinct frequencies, while Gallante et al.’s study^11^ lacked information in this regard. Moreover, there remains a need for further research to address the existing heterogeneity among studies and to assess the long-term implications of this method within the context of RIRS procedures.

Given the slight superiority observed with the lower pole displacement technique during RIRS, we emphasize the importance of conducting expanded research involving larger participant cohorts and mandatory prolonged follow-up periods^27^. This approach is essential for yielding more robust evidence favoring one technique over the other. Notably, our study is the first meta-analysis to compare these techniques. By incorporating additional studies, stronger evidence may be obtained, and even contribute to updates in guidelines.

## Conclusion

Through our study, we observed that displacing lower pole renal stones prior to lithotripsy results in a notable improvement in the SFR, while not causing any variance in laser energy consumption. Nonetheless, it’s important to note that this technique does come with the trade-off of prolonged surgical time. Despite these considerations, the advantages for patients undergoing this procedure are substantial, and it also offers benefits to surgeons in terms of enhanced ergonomics.

Given the limited size of the patient pool analyzed in this meta-analysis, it becomes imperative to conduct further research with larger and more diverse sample sizes. This will be essential in corroborating and reinforcing the findings of our study.

## Data Availability

The data will be available upon request.
